# The impact of bariatric surgery on asthma control differs among obese individuals with reported prior or current asthma, with or without metabolic syndrome

**DOI:** 10.1371/journal.pone.0214730

**Published:** 2019-04-09

**Authors:** Erick Forno, Peng Zhang, Mehdi Nouraie, Anita Courcoulas, James E. Mitchell, Bruce M. Wolfe, Gladys Strain, Saurabh Khandelwal, Fernando Holguin

**Affiliations:** 1 Department of Pediatrics University of Pittsburgh, Pittsburgh, Pennsylvania, United States of America; 2 Department of Medicine University of Pittsburgh, Pittsburgh, Pennsylvania, United States of America; 3 Department of Surgery University of Pittsburgh, Pittsburgh, Pennsylvania, United States of America; 4 Neutropsychiatric Research Institute, Fargo, North Dakota, United States of America; 5 Dept. of Surgery, Oregon Health and Science University, Portland, Oregon, United States of America; 6 Dept. of Surgery, Weill Cornell Medical College, New York, New York, United States of America; 7 Dept. of Surgery, University of Washington Medical Center, Seattle, Washington, United States of America; 8 Dept. of Medicine, University of Colorado, Denver, Colorado, United States of America; National and Kapodistrian University of Athens, SWITZERLAND

## Abstract

**Background:**

Both obesity and the metabolic syndrome have been independently associated with increased asthma morbidity. However, it is unclear whether metabolic syndrome limits the beneficial effects of weight loss on asthma.

**Objectives:**

To evaluate whether bariatric weight loss is associated with improved asthma control, and whether this association varies by metabolic syndrome status.

**Methods:**

We determined the changes in asthma control, defined by the Asthma Control Test (ACT), before and after bariatric surgery among participants with asthma in the multi-center Longitudinal Assessment of Bariatric Surgery (LABS) study, stratifying our analysis by the presence or absence of metabolic syndrome.

**Results:**

Among 2,458 LABS participants, 555 participants had an asthma diagnosis and were included in our analysis. Of these, 78% (n = 433) met criteria for metabolic syndrome (MetSyn) at baseline. In patients without MetSyn, mean ACT increased from 20.4 at baseline to 22.1 by 12–24 months, ending at 21.3 at 60 months. In contrast, among those with MetSyn there was no significant improvement in ACT scores. The proportion of patients without MetSyn with adequate asthma control (ACT >19) increased from 58% at baseline to 78% and 82% at 12 and 60 months, respectively, whereas among those with MetSyn, it was 73.8% at baseline, 77.1% at 12 months, dropping to 47.1% at 60 months (*p* = 0.004 for interaction between metabolic syndrome and time). Having MetSyn also increased the likelihood of losing asthma control during follow-up (HR = 1.92, 95% confidence interval [CI] 1.24–2.97, *p* = 0.003).

**Conclusions:**

Metabolic syndrome may negatively modify the effect of bariatric surgery-induced weight loss on asthma control.

## Introduction

Obese adults have higher asthma prevalence (11.1%) than normal (7.1%) or overweight (7.4%) adults. Asthma prevalence is even higher among obese women (14.6%) and obese non-Hispanic blacks (13.6%) [1]. Moreover, nearly 40% of asthmatics in the U.S. are obese [2]. Given that two thirds of the U.S. population is overweight or obese, this translates into millions of individuals with asthma and excess body weight and increased healthcare utilization [3]. There is growing recognition that obesity adversely impacts asthma; yet, the mechanisms are not fully understood [4]. For instance, it is unclear whether the association between these two conditions is mediated by excess weight and adiposity *per se*, or rather by the metabolic abnormalities that frequently complicate obesity. Approximately 60% of obese individuals meet metabolic syndrome criteria, which has been independently associated with increased asthma incidence, respiratory symptom severity, and lung function impairment [5–7]. Metabolic syndrome may independently affect airway function, and obese asthmatic patients with or without metabolic syndrome may represent different phenotypes.

Weight loss has been shown to improve asthma symptoms and severity [8–12]. However, most existing studies have been limited by small sample sizes and short follow-up times, and to date none have evaluated the role of concomitant metabolic syndrome. For this study, we hypothesized that improvements in asthma control or severity induced by weight loss through bariatric surgery would differ between obese asthmatics with and without metabolic syndrome. To test this hypothesis, we conducted a *post hoc* analysis on obese asthmatics participating in the Longitudinal Assessment of Bariatric Surgery (LABS) consortium, a large, NIH-funded, multicenter longitudinal study of adults undergoing weight loss surgery [13].

## Methods

### Study population

LABS Consortium Phase 2 (LABS-2) study participants with self-reported asthma diagnosis were selected for analysis. The details of LABS Consortium recruitment and procedures have been previously described [14–16]. Briefly, LABS is a cohort study of enrolled patients undergoing bariatric surgery in a consortium of 10 hospitals at 6 clinical centers. LABS-1 focused on the short-term (up to 30 days) safety of bariatric surgery using data collected on 5,108 participants. LABS-2 characterized the longer-term safety and health benefits of bariatric surgery and included 2,458 of LABS-1 participants, in whom the follow-up period was extended from 30 days to 6 years after surgery, with 6-month interval evaluations.

For our analysis, we collected the following variables from the longitudinal LABS-2 follow-up: asthma diagnosis (defined as “Have you ever been told by a doctor or other health professional that you have asthma”), age, sex, smoking (current, former, none), body weight, % body adiposity, race (White, Black, other) abdominal circumference, type of bariatric surgery, spirometry (done in a subset of patients as clinically indicated or per individual center’s protocol), asthma medication use (inhaled corticosteroids, long acting beta agonists, oral steroids, and other controllers), asthma control test (ACT) scores, which was ascertained annually along with triglycerides, high density lipoproteins (HDL), fasting blood glucose, and presence of comorbidities (diabetes, congestive heart failure, chronic obstructive pulmonary disease, hypertension, and sleep apnea).

### Metabolic syndrome

Following American Heart Association / National Heart, Lung, and Blood Institute guidelines, metabolic syndrome (MetSyn) was defined as having at least three of the following conditions: 1) abdominal obesity, defined as waist circumference ≥102 cm in men and ≥88 cm in women; 2) serum triglycerides ≥150 mg/dl or drug treatment for elevated triglycerides; 3) serum HDL <40 mg/dL in men and <50 mg/dl in women or drug treatment for low HDL; 4) blood pressure ≥130/85 mmHg or drug treatment for elevated blood pressure; 5) Hyperglycemia, defined as fasting plasma glucose ≥100 mg/dl (available only for the first 36 months of follow up), or drug treatment for elevated blood glucose [5].

### Asthma control

The Asthma Control Test (ACT) was administered at baseline and during each follow up visit, and used as a validated, five-item, self-administered questionnaire evaluating respiratory symptoms, use of rescue medications, daily function impairment secondary to asthma, and overall subjective level of disease control. Higher scores indicate better asthma control; in accordance with national guidelines, adequate asthma control was defined as having an ACT score >19 (and thus uncontrolled asthma as ACT ≤19)[17–20].

### Statistical analysis

Continuous variables were tested at baseline between patients with metabolic syndrome (MetSyn+) and those without (MetSyn-) using student’s t-test or Kruskal-Wallis; binary and categorical variables were tested by chi-square tests. Longitudinal analyses were performed using Generalized Estimating Equation (GEE) models to test the effect of bariatric surgery on different outcomes over time. In these models, we first tested the interaction between baseline MetSyn (pre-surgery) and visit month (during 0–60 months post-surgery) to assess the effect of pre-surgical MetSyn status on change of ACT score and on the proportion of patients with adequate asthma control. We also performed a time-lagged analysis to examine the association between MetSyn status at any visit with asthma control at the next visit. Finally, using a Cox survival analysis, we modeled the association between MetSyn and loss of asthma control at each visit, as well as with each MetSyn criteria separately. The robust variance estimator was used in all analyses. In addition, we performed a secondary analysis stratifying by type of bariatric surgery (RYGB vs. others). We handled the missing as non-informative and used complete case methods to analyze the data. All models were adjusted for potential confounders as appropriate, including age, race, gender and smoking status. All analyses were performed in Stata 14.2 (StataCorp., College Station, TX).

## Results

### Study population

Among the 2,458 subjects who participated in LABS-2, 607 reported a diagnosis of asthma [15]; of these, 52 were excluded due to insufficient data, leaving a total of 555 asthmatic participants for analysis. As shown in Table 1, 78% (n = 433) met criteria for metabolic syndrome at baseline (MetSyn+). Relative to those without metabolic syndrome (MetSyn-, n = 122 or 22%), MetSyn+ participants were older, more likely to be Caucasian, and more likely to smoke tobacco. MetSyn+ participants also had a higher rate of adequate asthma control at baseline (pre-surgery) than MetSyn- participants, although the median ACT score was not significantly different between the two groups. There were no differences in baseline anthropometric measurements, lung function, or asthma medication use between MetSyn+ and MetSyn- participants. Twenty seven percent of participants were lost to follow-up after the first year. These participants were younger (45 vs. 48 years old), less likely to be white (83% vs 90%), and more likely to have undergone gastric bypass surgery (82% vs 69%), but they were not significantly different in terms of MetSyn criteria, asthma control, or other characteristics (see S1 Table).

**Table 1:** Study population stratified by metabolic syndrome diagnosis at baseline.

	Absence of MetSyn (N = 122)	Presence of MetSyn (N = 433)^1^	P value
Age, median (IQR)^#^	44 (35–53)	48 (39–56)	0.003
Female Sex, n (%)	102 (84)	366 (85)	0.8
White Race, n (%)	98 (81)	386 (90)	0.009
Smoking, n (%)			0.012
Never	78 (64)	211 (49)	
Current	4 (3)	19 (4)	
Former	40 (33)	203 (47)	
Pack year, median (IQR)	0 (0–5)	0.1 (0–17)	<0.001
Weight (lb), median (IQR)	277(241–331)	286(254–325)	0.3
Body fat%, median (IQR)	52 (49–54)	52 (49–54)	0.6
Metabolic Syndrome:			
Waist circumference (cm), median (IQR)	128(117–141)	133(123–143)	0.08
Triglycerides (mg/dL), median (IQR)	100(77–128)	165(116–222)	<0.001
HDL (mg/dL), median (IQR)	54(46–61)	42(36–48)	<0.001
Hypertension, n (%)	57(47)	382(88)	<0.001
Hyperglycemia, n (%)	20 (16)	316 (73)	<0.001
Bariatric surgery:			
Gastric Bypass, n (%)	86 (70)	315 (73)	0.6
Other, n (%)	36 (30)	118 (27)	
Asthma:			
ACT score mean (S.D.)^##^	21 (4.0)	21 (4.0)	0.3
ACT score, median (IQR)	22 (18–24)	22 (19–24)	0.3
Asthma Controlled (ACT >19)^###^, n (%)	68 (61)	275 (74)	0.007
Use Asthma Medication, n (%)	54 (56)^2^	181 (51)^3^	0.3
Rescue inhaler, n (%)	38 (40)	109 (30)	0.09
ICS, n (%)	2 (2)	27 (8)	0.06
Anticholinergics, n (%)	0	3 (1)	0.9
Combination, n (%)	26 (27)	72 (20)	0.1
FEV1%, median (IQR)	83 (73–98)^4^	83 (66–92)^5^	0.6
FVC %, median (IQR)	83 (77–102)^4^	80 (70–92)^5^	0.2
FEV1/FVC % pred, median (IQR)	99 (90–103)^#^	100 (94–107)^##^	0.2
DLCO, median (IQR)	82 (74–90)^#^	81 (69–89)^##^	0.7

HDL: high-density lipoprotein; ICS: inhaled corticosteroids; FEV: forced expiratory volume in 1 second; FVC: forced vital capacity; DLCO: diffusing capacity of the lung for carbon monoxide.

^1^Presence of Metabolic syndrome = 433/555 (78%).

^2^n = 96.

^3^n = 355.

^4^n = 14–18.

^5^n = 53–88

^#^: Inter Quartile Range

^##^: Standard Deviation

^###^: Asthma Control Test

### Change in MetSyn after bariatric surgery

**Fig 1:**
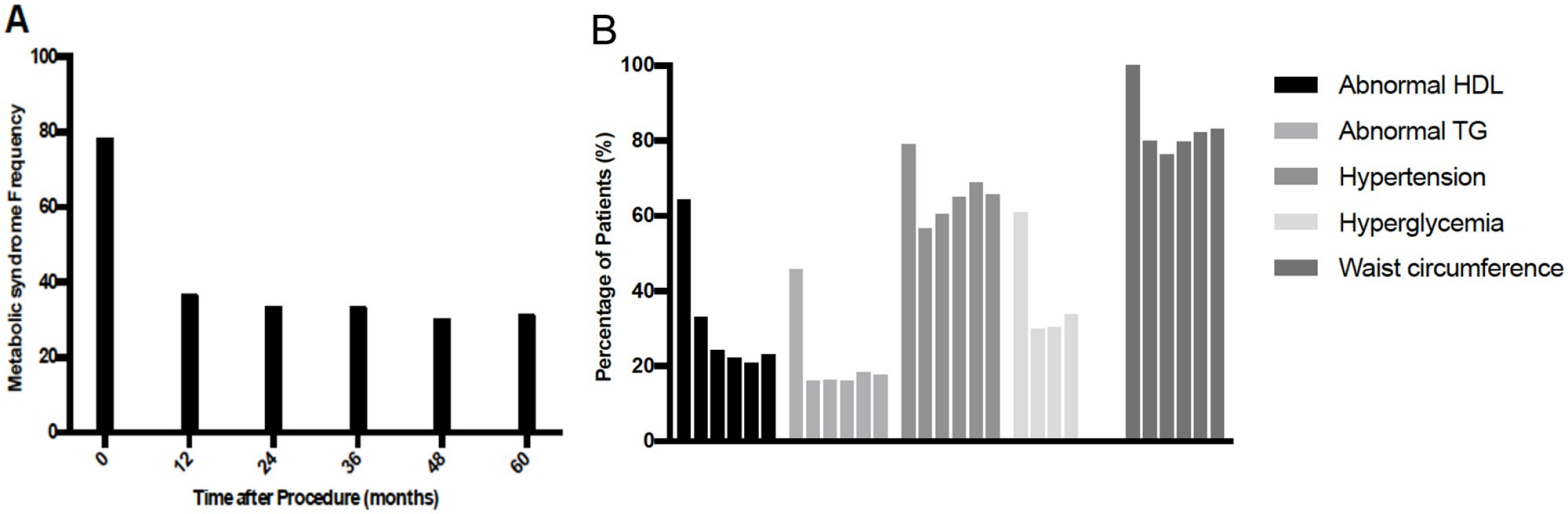
Metabolic syndrome and individual criteria at baseline and following bariatric surgery.

The proportion of participants with metabolic syndrome dropped from 78% at baseline to 36% at 1 year of follow-up, and subsequently continued to slowly drop to 30% by 60 months (Fig 1A). The changes of specific metabolic syndrome components are shown in Fig 1B: the proportion of patients with dyslipidemia (low HDL or high triglyceride levels) dropped by more than 50% and remained stable throughout the follow up period. There was a similar reduction in the prevalence of hyperglycemia. Reductions in the prevalence of hypertension or large waist circumference were relatively smaller.

### Change in weight after bariatric surgery

Participants experienced the greatest weight loss in the first 12–24 months after surgery, with a mean weight loss of 83–86 lbs (from a mean baseline weight of 291 lbs). At each visit, mean weight loss in the patients without MetSyn- at baseline was not significantly different from patients with MetSyn+ at baseline (p > 0.9, range of difference between two group -0.1–2.0 lbs) (S2 Fig).

#### Mean ACT score at baseline and average changes after bariatric surgery

**Fig 2:**
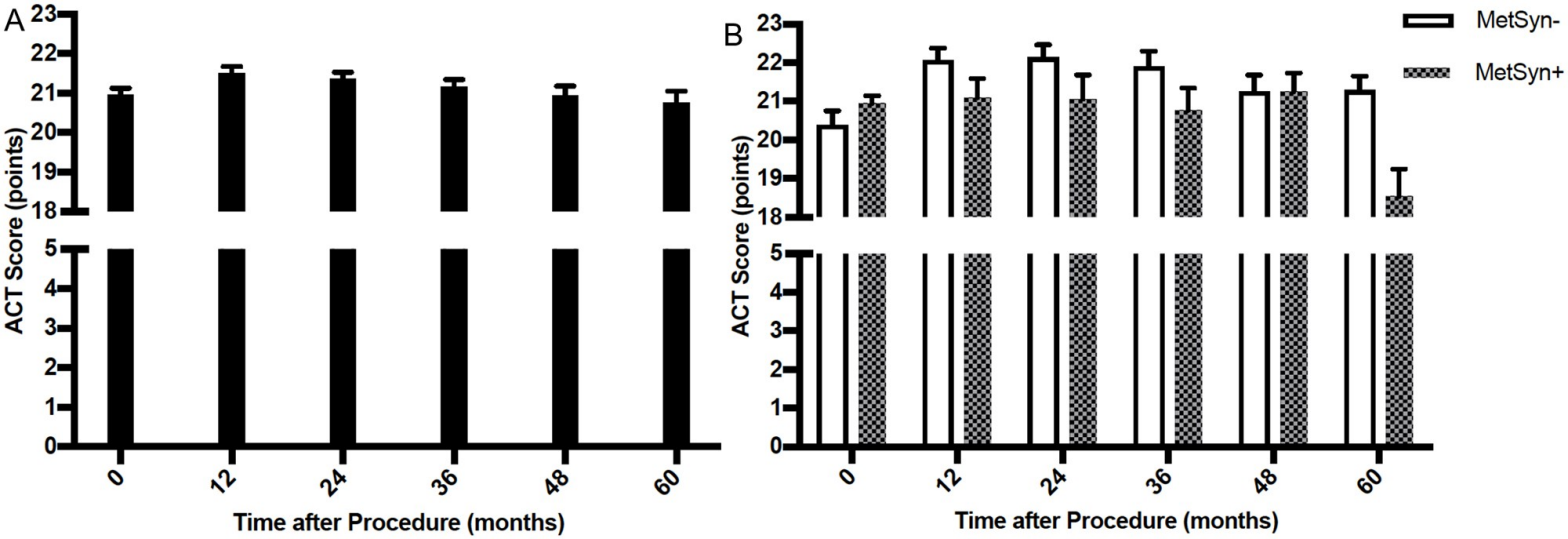
Mean ACT score at baseline and following bariatric surgery.

Overall, the mean ACT score initially increased (Fig 2A), reaching a peak of 21.5 points (SE = 0.17) 12 months after surgery before it gradually decreased to a mean of 20.8 (SE = 0.30) at 60 months, which was essentially the same as it was at baseline (*p* = 0.017 for change over time after adjustment for weight at each visit). When stratifying by MetSyn status and after adjusting for weight (Fig 2B), MetSyn at each visit was significantly associated with lower ACT score at the next visit (β = -1.2, *p* = 0.003 before weight adjustment and β = -1.1, *p* = 0.011 after weight adjustment).

#### Asthma control after bariatric surgery by MetSyn status, time-lagged analysis

**Fig 3:**
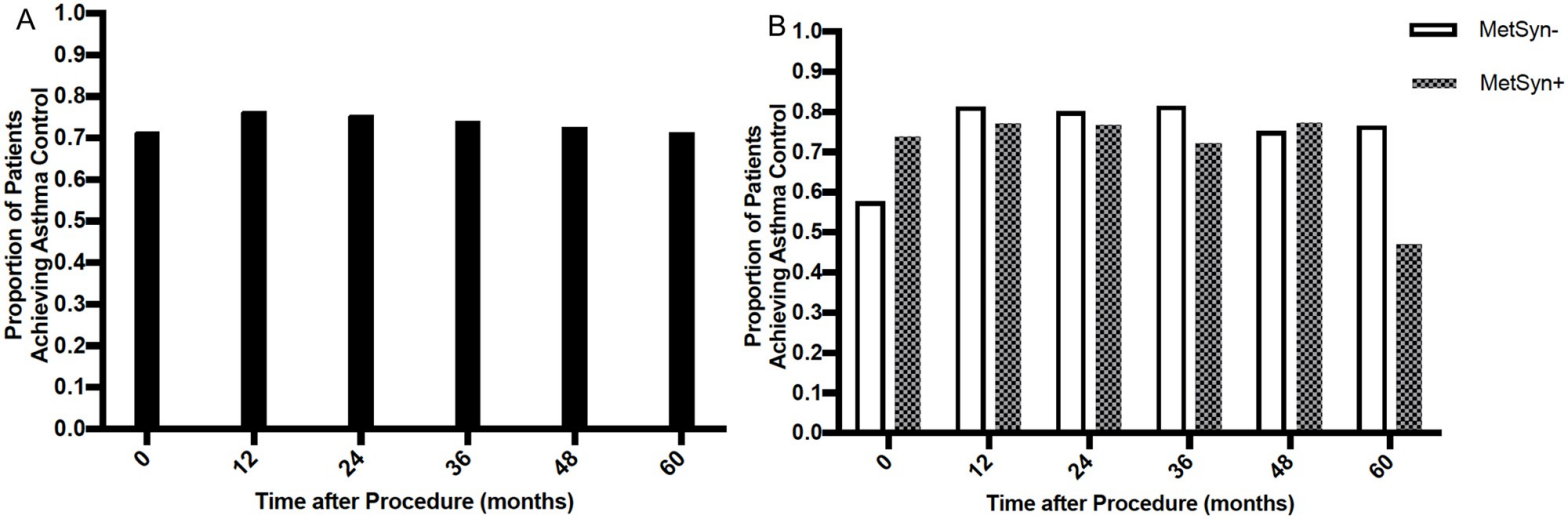
Proportion of patients achieving asthma control at baseline and following bariatric surgery.

The overall proportion of asthmatic patients having adequate asthma control was 71.5% at baseline (Fig 3A); this increased to 76.5% by 12 months after surgery and remained stable until 36 months before it gradually declined to 71.4% at 60 months (change in the proportion of asthma control over time *p* = 0.18). The proportion of MetSyn- patients with adequate asthma control increased from 58% at baseline to >80% at 12 months, and remained at 78% by 60 months (Fig 3B). In contrast, the asthma control rate among MetSyn+ participants remained largely unchanged from baseline through 48 months of follow up, and subsequently dropped to 47.1% at 60 months. In time-lagged analysis, the presence of MetSyn at each visit was also associated with lower odds of well-controlled asthma at the next visit (OR = 0.39, 95%CI = 0.21–0.73, *p* = 0.003 before weight adjustment and OR = 0.45, 95%CI = 0.23–0.86, *p* = 0.020 after weight adjustment).

### Asthma control after bariatric surgery by baseline MetSyn (pre-surgery) status

When comparing the proportion of controlled asthma based on baseline MetSyn status (S1 Fig), those with MetSyn+ had higher rates of adequate control before surgery (73% among MetSyn+ vs 61% among MetSyn-); however, they achieved a lesser improvement after the surgery (*p* = 0.009 for interaction between baseline MetSyn and follow-up time). Even after excluding the 60-month visit, the two groups still demonstrated a significant difference in asthma control rate (interaction *p* = 0.022). The difference in asthma control rate was not confounded by weight loss, as the relationship between MetSyn and asthma control remained statically significant after adjustment for it in the model.

### Asthma control by type of bariatric surgery

Most of the study participants underwent RYGB (72.3%), with relatively smaller numbers undergoing laparoscopic adjustable gastric band (22.7%), sleeve gastrectomy (3.2%), or another procedure (1.8%). Patients who underwent RYGB surgery had significantly greater improvement on ACT scores than those who underwent other types of bariatric surgery (mean change 0.944 vs 0.17, *p* = 0.001; adjusting for weight 0.91 vs 0.15 (p = 0.002)), but the proportion of patients achieving adequate asthma control was similar (*p* = 0.80; after weight adjustment p = 0.72).

### Loss of asthma control by MetSyn status

**Fig 4:**
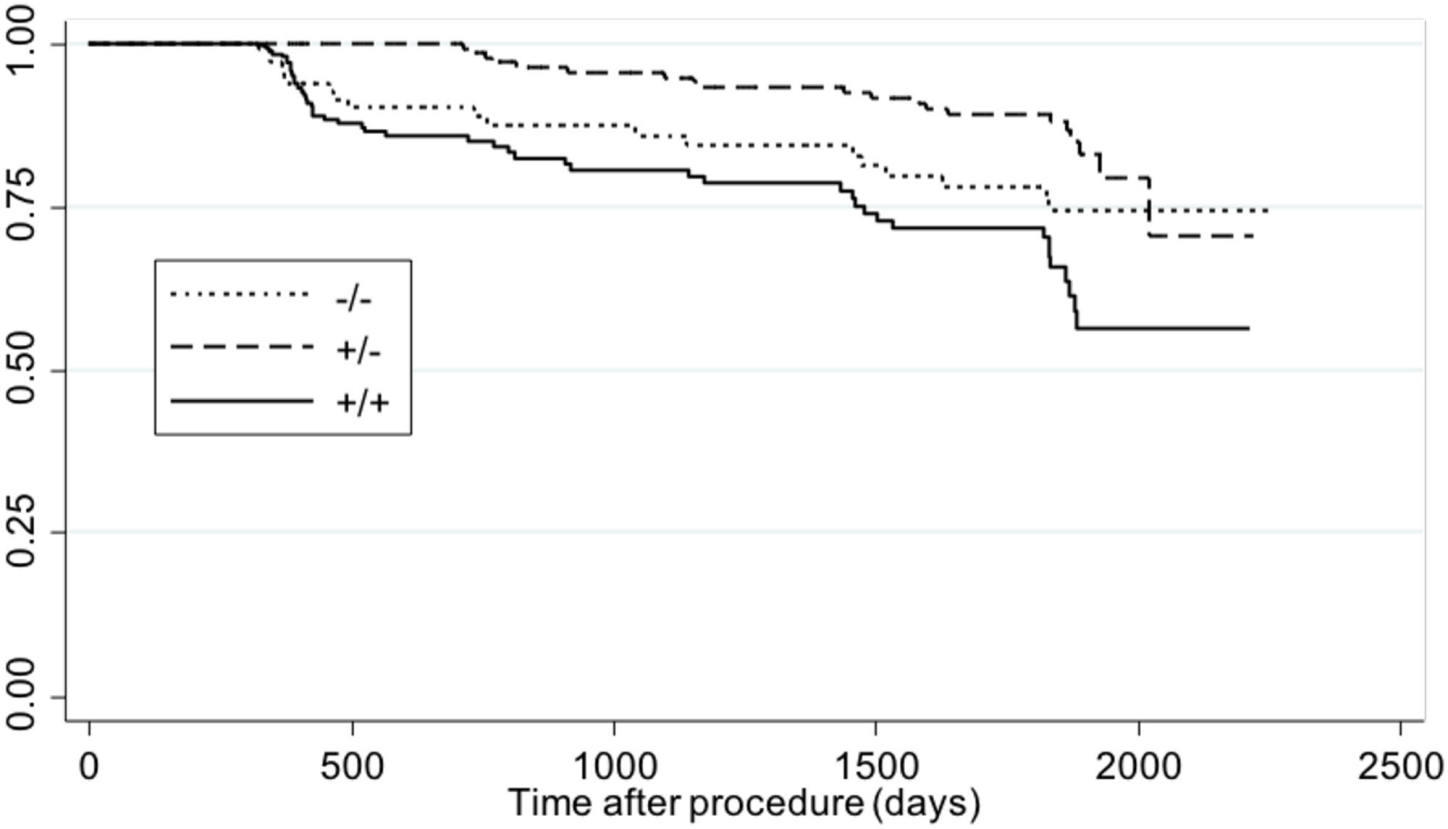
Adjusted Cox proportional hazard model for the loss of asthma control during follow-up, by presence or absence of metabolic syndrome.

To determine the relationship between MetSyn and the loss of asthma control after bariatric surgery, we used a Cox’s proportional hazards using MetSyn as a time-variant exposure (in order to account for changes in MetSyn status over time). Compared to participants that were MetSyn- both at baseline and during follow up (-/-), those with MetSyn+ at baseline and also during follow up (+/+) had the highest rate of losing asthma control throughout the study (Fig 4). After adjusting for sex, baseline smoking, and age, MetSyn was associated with a ~92% higher risk of loss of asthma control after bariatric surgery (hazard ratio [HR] = 1.92, 95% confidence interval [CI] = 1.24–2.97, *p* = 0.003). Among the individual components of MetSyn (Table 2), central obesity had the greatest impact on predicting loss of asthma control (HR = 2.74, 95%CI = 1.19–6.31, *p* = 0.013). Other significant risk factors included elevated triglycerides (HR = 1.81, 95%CI = 1.17–2.80) and low HDL (HR = 1.72, 95%CI = 1.13–2.62). Hyperglycemia and hypertension were not significantly associated with loss of asthma control.

**Table 2:** Association between the individual components of metabolic syndrome and the risk of uncontrolled asthma.

	HR (95% CI)	P value
Abnormal HDL	1.72 (1.13–2.62)	**0.012**
Abnormal TG	1.81 (1.17–2.80)	**0.007**
High blood pressure	1.24 (0.82–1.88)	0.30
Central obesity	2.74 (1.19–6.31)	**0.013**
Hyperglycemia	0.89 (0.57–1.40)	0.60
Metabolic syndrome	1.92 (1.24–2.97)	**0.003**
Bariatric surgery	0.85 (0.59–1.23)	0.40

HDL: high-density lipoprotein, TG: triglycerides

### Sensitivity analyses limited to patients with currently using asthma medications

In order to focus on those more likely to have current asthma, we conducted a sensitivity analysis of the study outcomes, limited to subjects with concomitant use of any asthma medications (227/534 = 43%). The results of this sub analysis were not different from the overall results. MetSyn at each visit continued to be significantly associated with lower ACT score at the next visit (β = -1.5, p = 0.014 after weight and season adjustment). In time-lagged analysis, the presence of MetSyn at each visit was also associated with lower odds of well-controlled asthma at the next visit (OR = 0.26, 95%CI = 0.09–0.71, p = 0.009 after weight and season adjustment). In the Cox proportional model, the Hazard Ratio (95% CI) for those with metabolic syndrome at baseline and during follow up (+/ +) vs those with metabolic syndrome at baseline but that lost it during follow up (+/ -) was 1.60 (1.15–2.23), p = 0.005, after adjustment for age, race, gender, season and smoking status Sensitivity analysis by baseline spirometry data.

Compared to LABS participants with spirometry (102/461), those without it had lower average baseline ACT scores (22 [95%CI 19–24) vs 21[95%CI 17–23), p = 0.01). However, the distribution of metabolic syndrome and its effects on ACT or asthma control did not differ across those with or without baseline lung function testing.

## Discussion

In this longitudinal analysis of obese adults with asthma undergoing bariatric surgery, there was a significant but transient improvement in the average ACT scores and in the proportion of participants achieving asthma control. To our knowledge, this is the largest study and longest follow up time to date evaluating the effects of bariatric surgery on asthma control in people with obesity and asthma.

The effects of bariatric surgery on asthma control have been evaluated in a small number of studies with limited numbers of patients. Maniscalco et al. reported that ACT scores increased from a mean of 18.7 to 22.2 (p<0.001) 1-year after having bariatric surgery, which is larger than the change we observed [8]. This difference could potentially be explained by the fact that their sample included only 12 mild obese asthmatic females, in contrast to our larger, more heterogeneous study population. Other studies have shown that within this time frame, the average asthma control questionnaire (ACQ) scores drop by more than 0.5, which is considered a clinically meaningful improvement [9, 12]. More recently, a large retrospective analysis of hospital databases reported that bariatric surgery in adults with asthma was associated with up to ~58% reduction in emergency department visits and hospitalizations for asthma up to 24 months after surgery [21]

Bariatric surgery can enhance asthma control through weight loss associated improvements in lung volumes, reductions in small airway resistance, and improvements in bronchial hyperresponsiveness. Yet, not everyone benefits equally, which suggests that other phenotypical factors, independently of the amount of weight loss, need to be considered [22].

We hypothesized that obese asthmatics with metabolic syndrome would benefit less from bariatric surgery than those without metabolic syndrome. Indeed, our results show that the proportion with asthma control during follow-up remained unchanged among participants with metabolic syndrome, and even dropped towards the end of the follow-up period. In contrast, those without metabolic syndrome had a large improvement after the first year and remained relatively unchanged for the remainder of time. Further, we show that participants with metabolic syndrome at baseline and during follow-up (i.e. their metabolic syndrome did not revert after the surgery) had the highest risk for losing asthma control at subsequent visits. These findings were statistically significant after adjusting for weight at each visit, suggesting that metabolic syndrome is associated with asthma control independently of weight loss. Coupled with studies showing that metabolic syndrome is associated with increased asthma incidence, greater morbidity and higher risk for airway obstruction and lung function decline, our results add to the cadre of studies causally linking metabolic syndrome to asthma [6].

There are several mechanisms that can potentially explain this association. Hyperinsulinemia can augment airway smooth muscle contractility and increase bronchial hyper-responsiveness, and abnormalities in glucose metabolism have been associated with greater risk for developing airway obstruction and steeper lung function decline over time [6, 23–26]. In addition, dyslipidemia has been associated with increased innate system activation and greater systemic inflammation [27, 28]. Abdominal adiposity has been linked to higher leptin, which in turn is associated with airway inflammation and greater risk for asthma [29]. Our results support the role for central obesity and dyslipidemia (reduced serum HDL and elevated triglyceride levels) as independent predictors of poor asthma control, which is similar to what others have reported [30, 31].

A unique aspect of this study is the ability to assess the association between asthma control and type of bariatric surgery. While the majority of the study population underwent RYGB, a third of them chose alternate procedures. Subjects that underwent RYGB lost more weight and had larger improvements in asthma control. Although subjects without MetSyn had larger improvements in asthma control outcomes in both the RYGB and non-GB surgeries, these differences were not statistically significant, possibly because of having smaller number of subjects in each comparison and consequently, insufficient power.

Our study has several limitations. This was a *post hoc* analysis of a study that was not designed specifically to assess asthma control, so some baseline characteristics are not matched, including the percentage of patients with controlled asthma in each group. A higher proportion of subjects in the metabolic syndrome group had well-controlled asthma at baseline, a potential ceiling effect (or the consequence of an unmeasured variable) that could explain why fewer could improve after surgery. However, our analysis also showed that patients whose metabolic syndrome failed to resolve after surgery had the greatest risk for losing control in subsequent visits, suggesting that the results are not exclusively determined by baseline group imbalances. Also, MetSyn + participants had higher use of ICS at baseline; which could reflect the fact that this group was more severe. However, this unbalance does not necessarily confound the association between changes in metabolic syndrome parameters and how bariatric surgery could have impacted asthma control during the follow up period. Rather, it highlights the importance of the problem. Moreover, the numbers of subjects on ICS were small and the differences were not statistically significant. The study also could not ascertain ICS adherence during follow up, so it is difficult to stipulate how these medications could have affected the study results. The diagnosis of asthma relied on patient-reported diagnosis, which can be inaccurate [32]. Study participants met criteria to participate in the LABS study and thus they may not be representative of all obese asthmatics undergoing bariatric surgery. All of these factors could limit the external validity of our results. The asthma case definition used in the LABS study does not differentiate between “ever” or “current” asthma; therefore, in order to focus on those more likely to have active disease, we conducted a sensitivity analysis limited to study subjects taking any asthma medications, and found that it did not change the results. In addition, the change in metabolic syndrome parameters and individual components of the ACT score among MetSyn+ participants, was not different in the time period between 48 and 60 months, when compared to all other time periods, and thus we are not able to explain the decline in asthma control at 60 months among this subgroup, nor did we have spirometry after surgery to evaluate how changes in lung function related to each group’s asthma control. We also did not have information on oral corticosteroid use to further assess asthma disease burden beyond ACT score. Numbers were too small to reliably analyze whether the type of bariatric surgery was an important factor in terms of subsequent asthma control. Finally, we are unable to quantify the degree of bias introduced by those who were lost to follow up; however, those lost to follow-up were not significantly different in terms of MetSyn criteria or asthma control, and we performed a sensitivity analysis by imputing those with metabolic syndrome status in missing follow-up, which did not significantly alter the results (data not shown).

In conclusion, asthma control improved following bariatric surgery in obese individuals; however, this may be a non-sustainable change that slowly tapers after the first year. Obese asthmatic individuals without metabolic syndrome are more likely to benefit in terms of asthma control from weight loss surgery. Further, the fact that the proportion of asthmatics with adequate asthma control improved in those without metabolic syndrome but remained unchanged to those with metabolic syndrome, along with the finding that metabolic syndrome was associated with the highest rate of loss of asthma control during follow up suggests that the effect of weight loss is less important in asthma control compared to metabolic syndrome. Although these findings need to be corroborated prospectively, it certainly warrants clinicians to consider controlling metabolic syndrome among obese subjects with asthma undergoing bariatric surgery. Among individual components of metabolic syndrome, central obesity and dyslipidemia were significantly associated with a greater risk for developing uncontrolled asthma after weight loss surgery.

## Supporting information

S1 Table Participants included vs. lost to follow-up at 12 months. (DOCX)

S1 Fig Proportion of patients achieving adequate asthma control at baseline and following bariatric surgery, by metabolic syndrome status at baseline. *p* = 0.009 for interaction between metabolic syndrome and time.
(TIF)

S2 Fig Weight loss following bariatric surgery by presence or absence of metabolic syndrome at baseline. Estimates adjusted for age, sex, type of surgery; p > 0.9 for differences in weight loss across metabolic syndrome groups.
(TIF)

## Abbreviations

ACT: Asthma Control Test
DLCO: Diffusion capacity of the lung for carbon monoxide
FEV1: Forced vital capacity in one second
FRC: Functional residual capacity
FVC: Forced vital capacity
HDL: High-density lipoprotein
ICS: Inhaled corticosteroids
LABS: Longitudinal assessment of bariatric surgery study
MetSyn: Metabolic syndrome
RYGB: Roux-en-Y gastric bypass
TG: Triglycerides
