# Supplementary material for: The impact of bariatric surgery on asthma control differs among obese individuals with reported prior or current asthma, with or without metabolic syndrome
Source: PLoS One. 2019 Apr 9;14(4):e0214730. doi: 10.1371/journal.pone.0214730 (PMC6456172; doi:10.1371/journal.pone.0214730)
Supplement: S1 Table — (DOCX) [file pone.0214730.s001.docx]

**Supplemental Table S1 – Participants included vs. lost to follow-up at 12 months**

|  | Lost to follow-up  (N = 149) | Followed-up  (N = 406) | P value |
| --- | --- | --- | --- |
| Age, median (IQR) | 45 (35-54) | 48 (39-55) | 0.009 |
| Female Sex, (%) | 82% | 85% | 0.3 |
| White Race, (%) | 83% | 90% | 0.031 |
| Smoking, (%) |  |  | 0.4 |
| Never | 48% | 54% |  |
| Current | 5% | 4% |  |
| Former | 47% | 43% |  |
| Pack year | 0.5 (0-19) | 0 (0-11.3) | 0.16 |
| Weight (lb), median (IQR) | 283 (253-331) | 283 (250-323) | 0.9 |
| Body fat%, median (IQR) | 53 (50-54) | 52 (49-54) | 0.09 |
| Metabolic Syndrome: |  |  |  |
| Waist circumference (cm), median (IQR) | 130 (123-143) | 132 (122-143) | 0.9 |
| Triglycerides (mg/dL), median (IQR) | 140 (104-203) | 142 (102-204) | >0.9 |
| HDL (mg/dL), median (IQR) | 43 (36-53) | 43 (37-53) | 0.7 |
| Hypertension, (%) | 76% | 80% | 0.3 |
| Hyperglycemia, (%) | 58% | 62% | 0.4 |
| Bariatric surgery: |  |  |  |
| Gastric Bypass, (%) | 82% | 69% | 0.002 |
| Other, (%) | 18% | 31% |  |
| Asthma: |  |  |  |
| ACT score | 22 (19-24) | 22 (19-24) | 0.9 |
| Asthma Controlled (ACT >19), (%) | 73% | 70% | 0.6 |
| Use Asthma Medication, (%) | 50% | 53% | 0.6 |
| Rescue inhaler, (%) | 35% | 31% | 0.5 |
| ICS, (%) | 6% | 7% | 0.8 |
| Anticholinergics, (%) | 0.9% | 0.6% | 0.8 |
| Combination, (%) | 22% | 21% | 0.8 |
| FEV1%, median (IQR) | 87 (73-93) | 81 (65-95) | 0.4 |
| FVC %, median (IQR) | 81 (67-91) | 80 (71-97) | 0.6 |
| FEV1/FVC % pred, median (IQR) | 102 (96-104) | 99 (92-107) | 0.6 |
| DLCO, median (IQR) | 83 (74-90) | 79 (68-90) | 0.9 |
|  | | | |
