# Supplementary figures and images for: The impact of bariatric surgery on asthma control differs among obese individuals with reported prior or current asthma, with or without metabolic syndrome
Source: PLoS One. 2019 Apr 9;14(4):e0214730. doi: 10.1371/journal.pone.0214730 (PMC6456172; doi:10.1371/journal.pone.0214730)

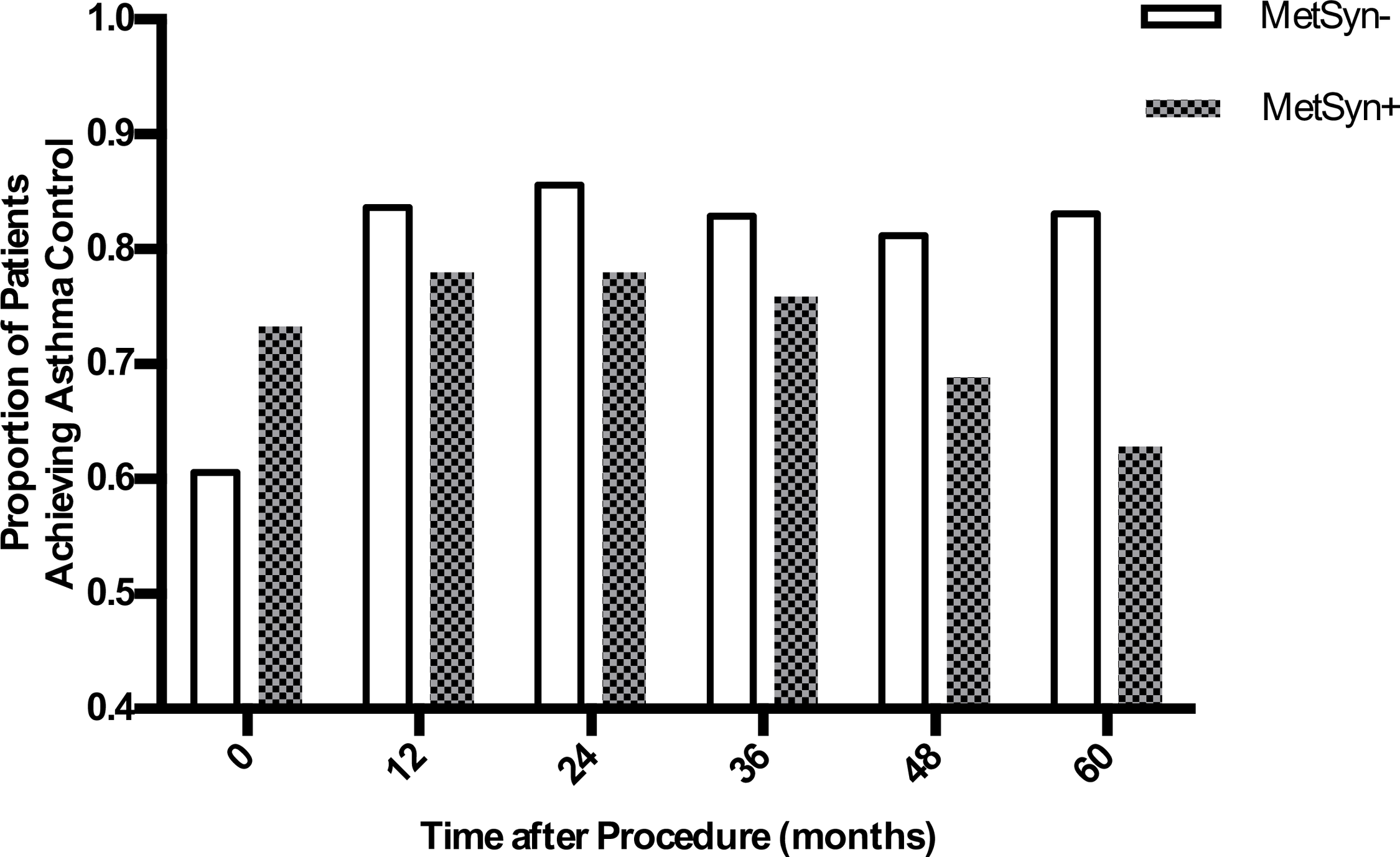

Supplement: S1 Fig — p = 0.009 for interaction between metabolic syndrome and time. (TIF) [file pone.0214730.s002.tif]

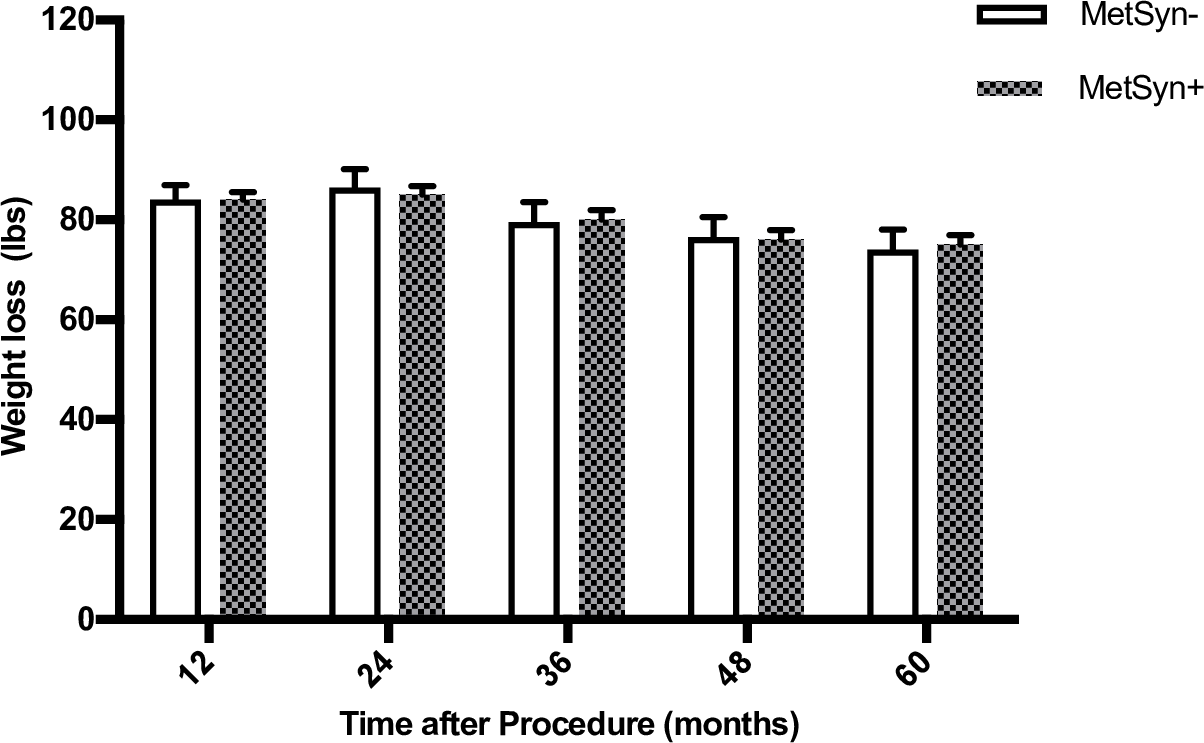

Supplement: S2 Fig — Estimates adjusted for age, sex, type of surgery; p > 0.9 for differences in weight loss across metabolic syndrome groups. (TIF) [file pone.0214730.s003.tif]
